# Commentary: Revisiting the Marshmallow Test: A Conceptual Replication Investigating Links Between Early Delay of Gratification and Later Outcomes

**DOI:** 10.3389/fpsyg.2018.02719

**Published:** 2019-01-10

**Authors:** Gladys Barragan-Jason, Cristina M. Atance, Astrid Hopfensitz, Jonathan Stieglitz, Maxime Cauchoix

**Affiliations:** ^1^Institute for Advanced Study in Toulouse, Toulouse, France; ^2^Faculty of Social Sciences, School of Psychology, Ottawa, ON, Canada; ^3^Toulouse School of Economics, Toulouse, France; ^4^SETE, Moulis, France

**Keywords:** patience, delay of gratification, social norm, children, behavioral plasticity

Given that “patience” is a recommended means to attain academic, social, and economic success (Mischel et al., 2010), efforts to promote the cognitive and behavioral traits underlying this ability early in life are warranted. While children's ability to be patient predicts future success (Mischel et al., 2010), a recent paper by Watts et al. (2018) suggests that the relationship between patience and success is not straightforward. Using a larger sample and a more sophisticated statistical approach than previous studies, they show that correlations between patience and future outcomes are not as strong as previously assumed, and that this association disappears after controlling for confounding factors (i.e., early social environment and demographic characteristics). Thus, to develop effective interventions, Watts et al. recommend reconsidering the actual measure of patience and its “broader cognitive and behavioral abilities” (in Watts et al., 2018, p. 17). We agree with Watts et al. and, to further this discussion, we raise two key questions regarding research on patience: (1) What, exactly, is patience? and (2) By what mechanisms does patience lead to successful outcomes?

## A Broader Definition of Patience

Across disciplines (e.g., Mischel et al., 2010; Hakimi and Hare, 2015), patience refers to the ability to delay gratification (Stevens and Stephens, 2008). Patience is thus evaluated with “delay tasks,” in which individuals choose between a smaller, immediately available reward (e.g., one candy, or $5), and a larger, delayed reward (e.g., two candies after 15 min, or $15 1 month later). However, this measure of patience (i.e., delay task) is narrow in scope because it excludes numerous factors such as social context and social trust recently shown to impact decision-making (Kidd et al., 2013; Michaelson et al., 2013; Higgs, 2015; Michaelson and Munakata, 2016; Barragan-Jason et al., 2018; Doebel and Munakata, 2018; Ma et al., 2018), as well as other factors related to the use of a reward including reward presentation (e.g., Imuta et al., 2014) and motivation for the reward (Paglieri et al., 2015). With respect to the latter, recent neurophysiological studies have highlighted the critical role of reward valuation (i.e., reward positivity) in affecting cognitive control (Cherniawsky and Holroyd, 2013; Schmidt et al., 2017). Delay tasks also fail to consider those instances during which one must wait without a “tangible” reward forthcoming (e.g., waiting your turn in the doctor's office; Barragan-Jason and Atance, 2017; Barragan-Jason et al., 2018). These instances are commonplace in daily life and often involve a social component.

Here, we argue that patience, which we define as the “ability to tolerate delay” (Barragan-Jason et al., 2018), is a broader concept than simply choosing whether or not to delay for a larger reward. Rather, this concept also encompasses behavioral (e.g., how we wait) and contextual (e.g., social/non-social; with/without gratification) factors that influence whether or not an individual displays patience (Barragan-Jason et al., 2018). In this vein, we developed the “pure waiting” (PW) task (Barragan-Jason and Atance, 2017) in which we measured children's patient behaviors (e.g., remaining still/static, playing/talking to oneself) when there was no tangible reward forthcoming. Patient behaviors in this task were correlated with performance in a delay task, suggesting that both tasks measure common underlying mechanisms (Barragan-Jason et al., 2018). Children also adjusted their patient behaviors by remaining significantly more static and playing/talking to themselves less when an experimenter was present vs. absent, highlighting the importance of social context in expressions of patience (Barragan-Jason et al., 2018). In sum, the PW task may better assess the influence of behavioral (“how we wait”) and contextual (social manipulations) factors than traditional delay tasks, and is a promising means of studying how individual differences in patience affect future outcomes.

## Social Norm Internalization as a Key Mechanism

Watts et al. suggest that “unobserved factors underlying children's delay ability may have driven long-run correlations” (in Watts et al., 2018, p. 17). Indeed, the idea that patience is uniformly beneficial has recently been questioned (e.g., Uziel, 2018). Individuals with high levels of patience fail to show behavioral flexibility (e.g., amoral behavior) when the context so requires (Denson et al., 2017). Moreover, patience is more malleable than previously assumed (e.g., Lee and Carlson, 2015), with performance being modulated by social context (e.g., Barragan-Jason et al., 2018; Doebel and Munakata, 2018) and environmental stability (e.g., Kidd et al., 2013; Michaelson and Munakata, 2016). An intriguing possibility is that internalizing patience as a social norm during childhood involves adjusting one's level of patience depending on social contexts (Schmidt and Rakoczy, 2016). The concept of social norm internalization is similar to what behavioral ecologists call “social plasticity” (i.e., an individual's ability to optimize its behavior depending on social context) which is associated with biological fitness in non-humans (e.g., Taborsky and Oliveira, 2012).

Consequently, our second argument is that it is not higher levels of patience, *per se*, that is key to explaining why patient children become more successful adults but, rather, a greater capacity to internalize behaviors as social norms during childhood. Specifically, successful adults may better internalize patience and other important abilities (e.g., cooperation: Gavrilets and Richerson, 2017; and altruism: McAuliffe et al., 2017) as social norms, during childhood. This ability, in turn, allows them to flexibly adjust those behaviors to different social situations throughout life.

In conclusion, Watts et al.'s (2018) study underscores the need to evaluate patience in a broader context. We advance this argument by providing a new perspective on patience and recommend that its measurement takes into account relevant behavioral and contextual factors. In so doing, we highlight the potentially critical role of social norm internalization when considering the predictive relationship between patience and long-term success. Future research directions, including the exploration of individual plasticity in patience across various social contexts (Taborsky and Oliveira, 2012) and its association with key social behaviors (e.g., cooperation, altruism, spite), will shed light on the possible role of children's social norm internalization in affecting their future success.

## Author Contributions

GB-J and CA drafted the manuscript. AH, JS, and MC provided critical revisions. All the authors approved the final version of the manuscript for submission.

### Conflict of Interest Statement

The authors declare that the research was conducted in the absence of any commercial or financial relationships that could be construed as a potential conflict of interest.
